# The Impact of the Hemoglobin-to-Lactate Ratio (HLR) on Clinical Outcomes and Prognosis in Pneumonia Patients Presenting to the Emergency Department

**DOI:** 10.3390/diagnostics16101508

**Published:** 2026-05-15

**Authors:** Fatih Ikiz, İlknur Şahin

**Affiliations:** Department of Emergency Medicine, Konya Beyhekim Training and Research Hospital, Konya 42130, Turkey; drilknurglshn@gmail.com

**Keywords:** biomarkers in pneumonia, hemoglobin-to-lactate ratio, HLR, lactate, hemoglobin, prognosis

## Abstract

**Background/Objectives:** Pneumonia remains a leading cause of emergency department visits worldwide, requiring rapid and objective risk stratification. While traditional scoring systems like CURB-65 and the Pneumonia Severity Index (PSI) are well-established, there is a constant need for dynamic biomarkers reflecting the underlying pathophysiology. This study aims to investigate the prognostic value of the hemoglobin-to-lactate ratio (HLR) in predicting mortality among pneumonia patients. **Methods:** This retrospective cohort study included 183 adult patients diagnosed with pneumonia at a tertiary training and research hospital between October 2024 and November 2025. Demographic data, clinical findings, laboratory parameters, and prognostic scores (CURB-65, PSI) were recorded. The impact of HLR on mortality was evaluated using univariate and multivariate logistic regression, while its predictive performance was assessed via Receiver Operating Characteristic (ROC) analysis and compared with clinical scores using DeLong’s method. **Results:** The overall mortality rate was 32.8%. HLR values were significantly lower in the exitus group compared to survivors (4.68 vs. 6.92, *p* < 0.001). Multivariate analysis revealed that an HLR ≤ 5.65 was an independent predictor of mortality, associated with a 10-fold increase in risk (OR: 10.0; 95% CI: 4.15–24.19; *p* < 0.001). HLR demonstrated high predictive power (AUC = 0.802), comparable to CURB-65 (AUC = 0.807) and PSI (AUC = 0.829). Notably, the combined HLR + CURB-65 model provided the highest diagnostic accuracy (AUC = 0.857, *p* = 0.037). **Conclusions:** HLR is a low-cost and easily accessible biomarker for predicting mortality in pneumonia. It effectively reflects the physiological balance between tissue oxygenation and metabolic failure. Integrating HLR into clinical practice, particularly when combined with traditional scores, can enhance risk (decision of discharge, admission unit [ward, ICU], evaluation of prognosis) in the emergency department.

## 1. Introduction

Pneumonia is defined as an infection of the distal lower respiratory tract, primarily involving the alveolar space, including the small bronchi and bronchioles. Globally, pneumonia is a clinical condition associated with high morbidity and mortality rates; consequently, rapid diagnosis and effective prognostic assessment are of vital importance, particularly for patients requiring critical care [1,2]. In recent years, rather than relying on individual biomarkers to predict clinical progression, composite ratios that simultaneously assess tissue hypoxia and the inflammatory response have gained prominence. The hemoglobin-to-lactate ratio (HLR) has emerged as a significant area of research for determining survival outcomes and disease severity in patients with pneumonia.

HLR is a proportional value that combines two critical variables representing the balance between the body’s oxygen delivery and both metabolic stress and tissue hypoxia at the cellular level (Lactate). Hemoglobin is the primary component in the transport of oxygen to the tissues [3]. Anemia or low hemoglobin levels developing during pneumonia further exacerbate already impaired gas exchange, thereby deepening tissue hypoxia. Lactate, on the other hand, is a classic indicator of inadequate tissue perfusion and anerobic metabolism [4]. High lactate levels are a harbinger of pneumonia-related sepsis or septic shock. In patients with pneumonia, a decrease in the HLR (low hemoglobin and/or high lactate) indicates the imbalance in tissue oxygenation and metabolic disruption. Considering these data in the literature, it has been suggested that the proportional relationship between hemoglobin and lactate may be a significant prognostic factor.

In patients with pneumonia, hemoglobin and lactate levels typically change in opposite directions, yet both trends reflect the patient’s deteriorating clinical status. During the course of pneumonia, a downward trend in hemoglobin levels is observed due to mechanisms such as systemic inflammation, cytokine storm and anemia of chronic disease: this condition further compromises the already limited oxygen supply to the tissues by reducing the oxygen-carrying capacity of the blood [5]. On the other hand, impaired gas exchange due to infection in the lungs and systemic perfusion disorders lead to cellular oxygen deprivation. This in turn causes the body to shift to anerobic metabolism for energy production [6]. As a direct result of this metabolic derangement, blood lactate levels begin to rise. This elevation in lactate becomes significantly more pronounced in cases progressing toward sepsis or septic shock [7]. As a result, as the severity of pneumonia increases, the concomitant decrease in hemoglobin and rise in lactate level creates a perilous combination that exacerbates tissue hypoxia. This synergy represents one of the fundamental pathophysiological shifts that reduces the patient’s chances of survival.

There are studies in the literature investigating the relationship between lactate and hemoglobin in critically ill patients [8]; to our knowledge, there is no existing study specifically exploring the prognostic properties of the HLR in patients with pneumonia. There remains a notable gap in the literature regarding the relationship between hemoglobin-to-lactate ratio (HLR) and prognostic measures, such as admission or discharge evaluation, CURB-65 scores, and the Pneumonia Severity Index (PSI). Furthermore, the effect of HLR on patient survival and its potential utility in initial emergency department assessment for individuals with pneumonia has yet to be fully elucidated. This study represents the initial investigation in the literature examining the impact of HLR levels on patients diagnosed with pneumonia, thereby offering a valuable addition to current scholarly research.

## 2. Materials and Methods

### 2.1. Research Model

Ethical approval for this study was granted by the KTO-Karatay University Ethics Committee, Konya, Turkey. (Decision No: 2025/051, Approval Date: 1 December 2025). Permission for the conduct of the study and data collection was obtained from the Education Planning Committee (EPC) of Konya Beyhekim Training and Research Hospital, approved on 3 December 2025 under decision number 01-01. Given the retrospective nature of the study, the requirement for individual patient informed consent was waived.

This is a retrospective cohort study, examining patient groups diagnosed with pneumonia who presented to the emergency department between 21 October 2024 and 21 November 2025. Patients aged 18 years and older, diagnosed with pneumonia and who were not pregnant were included in the study. Patients with a history of gastrointestinal bleeding were excluded from the study. Data collected included presenting complaints, clinical and thoracic CT results, comorbidities, vital signs, and laboratory results at first admission such as full blood count, biochemistry, arterial blood gas and hormones. Sociodemographic details for PSI score calculation were also included. Patient outcomes, including survival rate (mortality), admission unit assignment, length of stay and prognostic indicators during the follow-up period, were evaluated utilizing the Hospital Information Management System (HIMS) and epicrisis records. To reduce potential errors and achieve accurate calculations, prognostic scores for CURB-65 [9] and the PSI [10] were calculated with high precision using the proprietary computational tools developed internally by our team. Patients diagnosed with pneumonia were assessed using a combination of International Classification of Diseases (ICD) diagnostic codes, consultation notes and epicrisis reports. All data were fully anonymized during the collection phase. An initial dataset comprising 340 patient records was obtained; however, individuals lacking hemoglobin or lactate measurements (special circumstances such as insufficient or clotted blood samples), those without arterial blood gas analyses (e.g., clotted blood sample) or cases with incorrect ICD codes (patients diagnosed with other diseases irrelevant to pneumonia), or patients with significant secondary illnesses on admission that could potentially affect the results, such as gastrointestinal bleeding, were excluded. The final analysis therefore included 183 patients.

In this study, advanced statistical techniques were used to assess the association between HLR values and relevant prognostic factors. Correlations with inflammatory parameters, including C-reactive protein, procalcitonin, and white blood cell count, as well as CURB-65 and PSI were systematically evaluated through stratified modeling. The predictive capacity of HLR was determined both independently and alongside other prognostic indicators. Additionally, potential covariates such as age, sex, comorbidities and albumin were considered, and appropriate adjustments were made to ensure the objectivity of the findings.

### 2.2. Sample Size

To test the primary hypothesis, the use of independent *t*-tests was anticipated. The required sample size for the study was calculated using the G*Power (version 3.1) software (two tailed, effect size *d* = 0.5 (moderate), α = 0.05). It was determined that a minimum sample size of 128 was required to achieve 80% power. The effect size levels were determined based on Cohen’s *d* recommendations [11]. This study was completed with a final sample size of 183. When evaluated via post hoc power analysis, the final power of our study was recorded as 88.5% (critical t = 1.97).

### 2.3. Statistical Methods

Statistical analyses in the study were performed using the SPSS 31.0 (IBM Inc, Chicago, IL, USA) software. The Kolmogorov–Smirnov test, histogram analyses, skewness/kurtosis data, and Q-Q plots were considered to evaluate the normality of continuous variables. Continuous variables were expressed as median [range: minimum–maximum]) or mean ± standard deviation. Qualitative data are expressed as frequency (*N*) and percentage (%). Relationships of continuous variables between two groups were examined using the Mann–Whitney U test or the independent-samples *t*-test. The effect profiles of variables on mortality were evaluated with univariate and multivariate logistic regression analysis (with a limitation of 10 events per variable). Box–Tidwell and multicollinearity assumptions were controlled prior to multivariate logistic regression analysis. Receiver operating characteristics (ROC) analyses were carried out to determine cut-off values in terms of binary outcomes. Optimal cut-off values are determined based on Youden’s J index. The models were obtained and calculated using probability scores resulting from logarithmic pooling via logistic regression. Stratified models were built with ROC analysis and the predictive values for models are compared using DeLong’s method. Relationships between qualitative variables were investigated with Pearson’s chi-square or Fisher’s exact tests. Correlations between continuous variables are investigated with Pearson’s or Spearman’s correlation analysis, and the results are summarized with a correlogram chart. In the entire study, the type-I error rate was set at 5% (α = 0.05), and a *p*-value of less than 0.05 was considered statistically significant.

## 3. Results

Our study was completed with a sample of 183 participants, and the statistical distribution characteristics of the quantitative data for the general sample are summarized in Table 1.

Gender, sociodemographic characteristics, comorbidities, and clinical and radiological findings are summarized in Table 2. In our study, pneumonia patients were predominantly male (56.8%), and it was observed that 150 (82.0%) patients had at least one comorbidity. While the most common comorbidity was chronic obstructive pulmonary disease (COPD) at 60.7%, this was followed by hypertension (HT) at 43.7%, renal failure at 30.6%, and diabetes at 29.0%, respectively. On the other hand, 151 patients (82.5%) were hospitalized, and 62.3% of these patients were admitted to the ICU. Furthermore, 60 patients (32.8%) were noted as exitus (mortality) during the follow-up period. The number of patients diagnosed with sepsis was recorded as 139 (76.0%).

Distribution of gender, comorbidities, pneumonia, and clinical characteristics are compared among survival groups. In the analysis, while the presence of comorbidity was noted to be higher in the exitus group (90% vs. 78.05%, *p* = 0.048), the distribution rates of cerebrovascular disease (*p* = 0.002), renal failure (*p* = 0.003), Alzheimer’s disease/Dementia (*p* = 0.003), hypertension (HT) (*p* = 0.032), hyperlipidemia (HL) (*p* = 0.03), and coronary artery disease (CAD) (*p* = 0.017) were found to be significantly higher in the exitus group compared to the survival group. As expected, the rates of ICU admission, erythrocyte suspension (ES) therapy, vasopressor support, septicemia, and dialysis were significantly higher in the exitus group. Additionally, the rates of pleural effusion (*p* = 0.002) and ground-glass opacities (*p* = 0.006) were found to be higher in the exitus group, and all findings and distributional ratios are summarized in Table 3.

**Figure 1:**
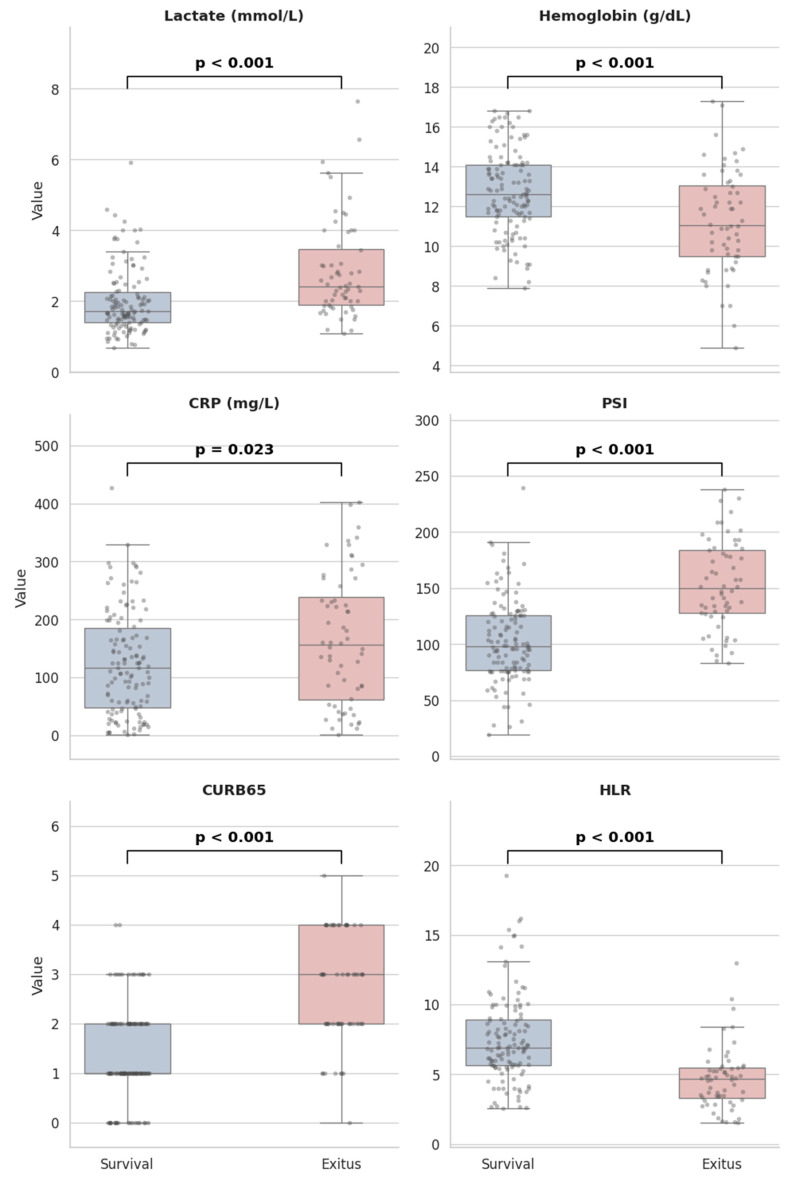
Boxplot summaries of some prognostic and laboratory variables showed significant differences between groups (Abbreviations: CRP = C-reactive protein, PSI = Pneumonia Severity Index, HLR = hemoglobin-to-lactate ratio).

Laboratory results, vital signs, and clinical features are compared among survival groups. In the analysis, age (*p* < 0.001), respiratory rate (*p* = 0.001), heart rate (*p* = 0.002), pCO2 (*p* = 0.021), lactate levels (*p* < 0.001), BUN (*p* < 0.001), blood glucose (*p* = 0.014), RDW (*p* = 0.011), CRP (*p* = 0.023), urea (*p* < 0.001), creatinine (*p* = 0.003), ICU length of stay (LOS) (*p* = 0.001), total LOS (*p* = 0.003), CURB-65 (*p* < 0.001), and PSI (*p* < 0.001) scores were found to be statistically significantly higher in the exitus group. On the other hand, diastolic blood pressure (DBP) (*p* = 0.006), oxygen saturation at initial admission (pulse oximetry) (*p* = 0.046), pH (*p* < 0.001), hemoglobin (*p* < 0.001), albumin (*p* < 0.001), and GCS (*p* < 0.001) scores were found to be significantly lower in the exitus group. In the study, while the median lactate level was 2.42 mmol/L in the exitus group, it was noted as 1.72 mmol/L in the survival group (*p* < 0.001). On the other hand, hemoglobin levels were 11.25 ± 2.60 g/dL in the exitus group and 12.74 ± 2.10 g/dL in the survival group. Based on these data, it was concluded that particularly high lactate levels (>2 mmol/L) and anemia are associated with mortality (*p*-values < 0.001). When HLR values, which reflect the balance between increased lactate and decreased hemoglobin, were examined, they were found to be lower in the exitus group compared to the survival status (4.68 versus 6.92, *p* < 0.001). The results indicated a marked elevation in inflammatory parameters including C-reactive protein (CRP) and red cell distribution width (RDW). However, no statistically significant differences were identified in other inflammatory markers such as white blood cell count, neutrophils and procalcitonin. Also, it was noteworthy that prognostic indicators such as CURB-65 and PSI scores were significantly higher in the exitus group, as expected. Distributional comparisons of the quantitative data are summarized in Table 4. The distributions of lactate, hemoglobin, CRP, CURB-65, PSI, and HLR are summarized as boxplot graphs in Figure 1.

HLR levels were compared based on the presence of pleural effusion and other specific clinical circumstances (Table 5). In the comparison between patients with and without pleural effusion, albumin level was considered as a confounding factor that could contribute to the development of effusion and influence the outcomes. Accordingly, albumin levels were included in the analysis as a potential covariate, and statistical comparisons were performed with adjustments for this parameter. While independent analyses showed significant differences in HLR levels between the groups (*p*_1_ = 0.013), re-evaluation after adjusting for albumin demonstrated that HLR levels did not differ significantly according to pleural effusion status (*p*_2_ = 0.212). Our results indicate a significant causal relationship between pleural effusion and albumin levels, identifying albumin as a potential covariate influencing the clinical outcomes (pleural effusion). Once adjusted for the albumin, it was understood that HLR levels did not constitute a significant difference between the effusion groups (Table 6).

Correlation relationships between quantitative variables are summarized with a correlogram. Depending on the normal distribution characteristics of the data, Pearson (correlation coefficient: r) or Spearman (correlation coefficient: rho) analyses were utilized. In the analysis, a moderate negative significant correlation was observed between HLR and both CURB-65 and PSI scores (rho values were −0.457 and −0.499, respectively; *p*-values < 0.001). A significant moderate positive correlation was found between GCS and HLR values (rho = 0.444; *p* < 0.001). On the other hand, it was noted that age, CRP, and PCT levels had significantly weak correlations with HLR (*p*-values < 0.05).

**Figure 2:**
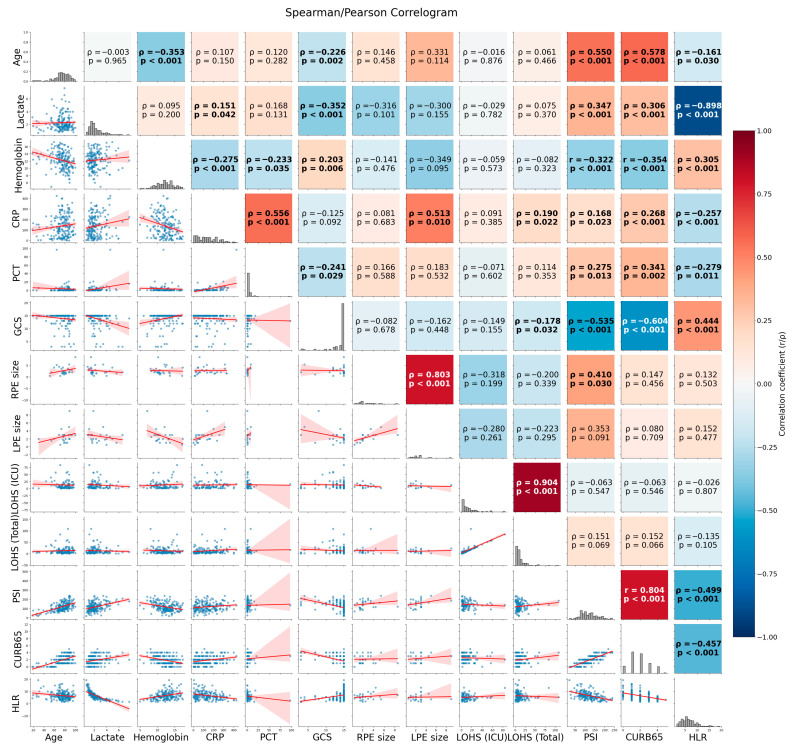
Summary of the correlation relationships between age, prognostic factors (inflammatory variables, ICU and total length of stay, pneumonia scores), hemoglobin, lactate, and HLR values using a correlogram (while Pearson correlation analysis was used for the correlation relationships between hemoglobin, PSI, and CURB-65, Spearman correlation analysis was used for the other correlation relationships) (Abbreviations: CRP = C-reactive protein, PCT = procalcitonin, GCS = Glasgow Coma Scale, RPE = right pleural effusion, LPE = left pleural effusion, LOHS = length of hospital stay, ICU = intensive care unit, PSI = Pneumonia Severity Index, HLR = hemoglobin-to-lactate ratio).

When other correlation relationships among the data were examined, a very strong correlation was observed between pneumonia prognostic indicators (CURB-65 and PSI) (r = 0.804; *p* < 0.001). On the other hand, GCS scores showed a significant moderate negative correlation with PSI (rho = −0.535; *p* < 0.001), while they showed a strong negative correlation with CURB-65 (rho = −0.604; *p* < 0.001). Furthermore, moderate positive correlations were noted between CRP levels and both PCT and left pleural effusion (LPE) dimensions (rho values were 0.556 and 0.513, respectively; *p*-values < 0.001). The correlogram plot and correlation relationships between other quantitative variables are summarized in Figure 2.

In the univariate logistic regression analysis, where variables were evaluated independently, it was observed that age, CRP, CURB-65, PSI, and HLR values had a significant impact profile on mortality. In the analysis, the group with age ≥ 77.0 noted a 3.91-fold increase in mortality compared to the <77.0 group (*p* < 0.001), while no significant regression relationship was noted for gender and comorbidities. The group with CRP ≥ 213.5 was found to be associated with a 2.7-fold increase in mortality compared to the reference category (*p* = 0.005). When prognostic scores were examined, the group with CURB-65 ≥ 2.0 was associated with a 9.18-fold increase in mortality compared to the reference category (*p* < 0.001), while an 11.33-fold increase in mortality was noted in the group with PSI ≥ 133.0 compared to the reference category (*p* < 0.001). When HLRs were examined, the group with ≤5.65 showed a 12.15-fold increase in mortality compared to the group with >5.65 (*p* < 0.001).

Variables that were significant in the univariate regression analysis were adjusted with a multivariate regression analysis. While PSI was excluded from multivariate analyses because it violated the assumptions of the analysis and did not meet the multicollinearity assumption, gender and comorbidities were included in the multivariate analyses for adjustment purposes as they were considered as potential covariates. Age maintained its significance; the group with age ≥ 77.0 was shown to be associated with a 4.13-fold increase in mortality compared to the <77.0 group (*p* = 0.002). On the other hand, it was noted that CRP levels lost their significance in multivariate analyses (*p* = 0.089). Looking at CURB-65 values, the group with a score ≥ 2.0 was associated with a 2.77-fold increase in mortality compared to the reference category, and its significant relationship with mortality was shown to continue (*p* = 0.04). Finally, when HLR levels were examined, the group with an HLR level ≤ 5.65 was found to be associated with 10 times the mortality compared to the group with >5.65 (*p* < 0.001) (Table 7).

**Figure 3:**
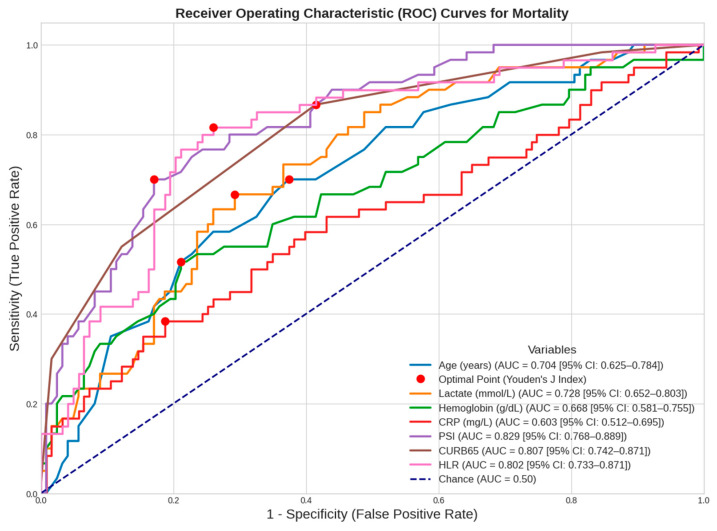
ROC analysis of variables in terms of mortality prediction.

The cut-off values and predictive characteristics of age, lactate, hemoglobin, CRP, PSI, CURB-65, and HLR values associated with mortality were investigated in detail. The ROC analysis revealed significant cut-off values for all variables. Notably, the AUC was >0.70 for the lactate, PSI, CURB-65, and HLR parameters. Detailed examination showed that the highest sensitivity and negative predictive values (NPVs) were observed in CURB-65, 86.7% and 90%, respectively, while the highest specificity and positive predictive values (PPVs) were found in the PSI score, 82.9% and 66.7%, respectively. It was understood that the PSI (AUC = 0.829), CURB-65 (AUC = 0.807), and HLR (AUC = 0.802) values, which had the highest AUC values, were the most valuable diagnostic variables (*p* < 0.001) (Table 8, Figure 3).

**Figure 4:**
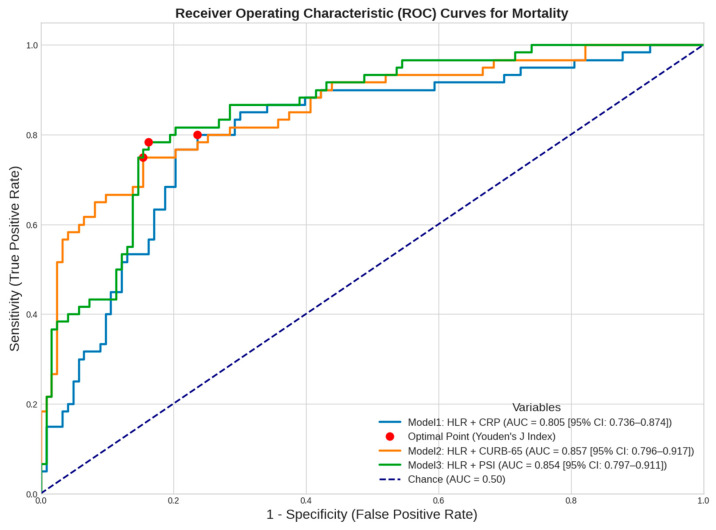
ROC analysis of combined models in terms of mortality prediction.

**Figure 5:**
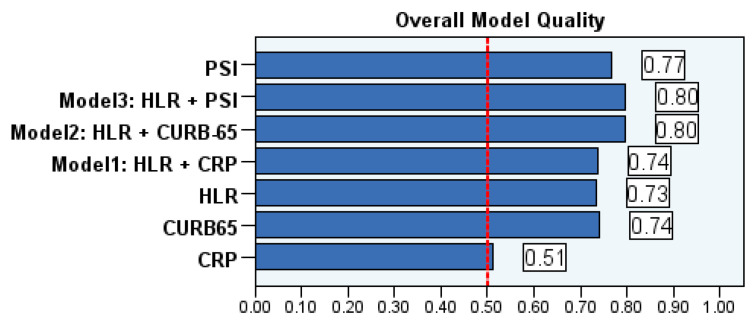
Overall quality of ROC analysis models: The overall quality values of the variables/models are presented, and the results indicate that the best predictive performance is achieved by Models 2 and 3. It was observed that PSI, HLR, CRP, and CURB-65 values alone remained behind the performance of Models 2 and 3. Furthermore, when considering DeLong’s method, Model 3 demonstrated significantly higher performance in pairwise comparisons compared to CRP and CURB-65 (*p* < 0.05). Upon examination of Model 2, it was found to perform significantly better than HLR, CURB-65, and CRP alone (*p* < 0.05). As for Model 1, it outperformed only CRP (*p* < 0.05), showing no significant performance advantage compared to the other variables individually (*p* > 0.05). Considering the overall results, it was concluded that Model 2 is the best model for mortality prediction due to its higher AUC (85.7%) values and the highest PPV (70.3%).

Three different models were established by combining HLR levels with CRP, an inflammatory marker, and prognostic scores CURB-65 and PSI. The models were constructed as Model 1: HLR+CRP, Model 2: HLR+CURB-65, and Model 3: HLR+PSI, and the direction of the diagnostic and predictive characteristics was investigated using these models. In the analysis, the AUC values in Model 2 and Model 3 increased to 85.7% and 85.4%, respectively, and were found to be superior compared to Model 1. On the other hand, to perform more objective comparisons between the models, AUC values were compared using DeLong’s method, and pairwise comparisons between models were conducted. Although Models 2 and 3 appear to be the best when considering AUC values, the pairwise comparison results indicate a statistically significant difference in Model 2, marking it as the most promising model (z = −2.081; *p* = 0.037). Although Model 3 had a higher AUC value compared to Model 1, the level of significance was nearly missed in the pairwise model comparisons (z = −1.903; *p* = 0.057) (Table 8 and Table 9, Figure 4). In addition to the standalone values of HLR, PSI, CURB-65, and CRP, the overall quality values from the ROC analysis of the combined models are presented in Figure 5.

Our results indicated that HLR levels are a valuable variable in predicting mortality, with 81.7% sensitivity, 74% specificity, 89.2% NPV, and 60.5% PPV.

## 4. Discussion

While pneumonia remains one of the most frequent reasons for emergency department presentations worldwide, predicting the clinical severity of patients and performing early risk stratification constitutes the cornerstone of therapeutic success [1]. Although traditionally utilized scoring systems such as CURB-65 and the Pneumonia Severity Index (PSI) are well established, there remains an ongoing need for more rapid, objective and pathophysiologically based biomarkers for clinicians managing a dynamic clinical course [12,13,14]. The primary finding of our study is that the hemoglobin-to-lactate ratio (HLR), which integrates the oxygen-carrying capacity of hemoglobin with the role of lactate as a marker of cellular hypoxia, serves as a highly potent tool for predicting mortality in patients with pneumonia. The results demonstrated that HLR values were significantly lower in the mortality (non-survival) group compared to survivors (4.68 vs. 6.92) and an HLR value below 5.65 corresponded to a 10-fold increase in mortality risk. These findings are consistent with studies in the literature examining similar pathophysiological mechanisms. A recent study conducted by Ağaçkıran et al. examined the lactate-to-hemoglobin ratio in non-traumatic critically ill patients across various diagnostic categories and found that this ratio was as effective as or even superior to established scoring systems for predicting mortality outcomes [8]. In the mentioned study, while lactate levels were significant prognostic indicators, it was demonstrated that the lactate-to-hemoglobin ratio was clinically more useful, showing higher values in mortality (AUC: 0.676 vs. 0.749; sensitivity = 70.1%, specificity = 71%; *p* < 0.001). Our findings are consistent with these results; however, compared to hemoglobin or lactate alone, our HLR values demonstrated higher AUC, sensitivity and specificity (AUC = 0.802, sensitivity = 81.7% and specificity = 74%; *p* < 0.001). A distinguishing feature of our study is the establishment of this correlation specifically within a cohort of pneumonia patients, utilizing correlations with key metrics such as CURB-65 and PSI. The higher c-statistic observed in our findings relative to those reported by Ağaçkıran et al. may be attributable to our targeted focus on pneumonia cases. We conclude that HLR demonstrated notable significance and clinical utility in this patient population.

The systemic inflammatory response and cytokine storm that develop in the pathophysiology of pneumonia can lead to a decrease in hemoglobin levels via the mechanism of anemia of chronic disease, either by suppressing erythropoiesis or by inducing iron sequestration. In our study, hemoglobin levels were found to be significantly lower in the exitus group (11.25 g/dL vs. 12.74 g/dL; *p* < 0.001), which aligns with the large-scale analysis conducted by Marziano et al. on more than 15 000 patients, which demonstrated that profound anemia is strongly associated with increased mortality rate in patients with community-acquired pneumonia (CAP) [5]. Low hemoglobin levels further restrict oxygen delivery to the tissues in patients whose gas exchange is already impaired at the alveolar level [15]. This restriction at the cellular level results in a shift toward anerobic metabolism and an increase in lactate. Chalmers et al. found that lactate levels are an independent severity marker in pneumonia patients [7]. Our data support this theory, showing significantly higher lactate levels in the mortal group at 2.42 mmol/L. The HLR integrates these two interrelated yet contrasting parameters into one measurement, representing the equilibrium between tissue oxygenation and metabolic stress. It provides a more robust prognostic insight than either lactate or hemoglobin alone. Considering both our study and the literature data, it is accurate to state that lactate and hemoglobin values are independent risk factors; furthermore, given the cause-and-effect relationship between them, carefully analyzing the balance between lactate and hemoglobin and utilizing an index (HLR) for this purpose is a crucial detail in clarifying this balance and directly predicting clinical prognosis.

Another significant outcome of this study is the moderate negative correlation observed between HLR and both CURB-65 (rho = −0.457) and PSI (rho = −0.499). This suggests that increased disease severity corresponds to lower HLR values, consistent with patterns reflected in these clinical scores. ROC analysis indicated an AUC for HLR (0.802) closely aligned with CURB-65 (0.807), implying that HLR, derived from a single blood test, may be a promising biomarker in evaluating patients diagnosed with pneumonia. Additionally, Model 2 (AUC = 0.857), which combines HLR with CURB-65, demonstrated enhanced predictive accuracy compared to HLR alone, affirming its status as the most effective statistical model. This aligns with the general trend in the literature, which emphasizes the supportive role of biomarkers in enhancing clinical scoring systems.

Furthermore, an interesting observation was noted regarding the inflammatory parameters. Although CRP levels were elevated in the mortality group and demonstrated significance in univariate analysis, this significance was lost in the multivariate analysis. This suggests that while CRP has high diagnostic value in pneumonia, its capacity to predict survival may be overshadowed by more dynamic variables such as age, CURB-65 and HLR. Our findings indicate that CRP may be influenced by potential covariates, such as age, inflammatory states and comorbidities, and may be associated with elevation mechanisms distinct from septicemia. Additionally, the absence of a significant difference in white blood cell count (WBC) and procalcitonin levels between the survival groups suggests that the prognostic power of these markers may be limited during the initial emergency department presentation. On the other hand, age continued to be among the most significant independent risk factors for mortality identified in our research, aligning with findings reported in all existing pneumonia studies. Specifically, patients aged 77 years and above exhibited an increase in mortality risk exceeding fourfold. In a research study conducted by Gonçalves-Pereira et al. on patients with community-acquired pneumonia, a significant correlation was demonstrated between increasing age and survival; specifically, a 4.37-fold increase in mortality was observed in the 66–80 age group (OR: 4.37 [4.05–4.71]), which aligns with our own findings [16]. While septicemia was found to be statistically significantly more prevalent in the mortality group (*p* < 0.001), the absence of significance for CRP values in multivariate logistic regression analysis, along with the overall non-significant findings for PCT levels, indicates that these biomarkers should not be regarded as definitive criteria for sepsis [17]. This serves as a significant point highlighting the necessity for further research into potential biomarkers for predicting prognosis in pneumonia in patients.

In this study, the ANCOVA technique that was applied to assess the relationship between pleural effusion and albumin levels represents a noteworthy methodological advancement within the field. While an initial association was observed between the presence of effusion and HLR, this significance was eliminated after adjusting for albumin levels, indicating that hypoalbuminemia serves as a common confounder in both the development of effusion and poor prognosis, resulting in low HLR. The results highlight the importance of evaluating albumin levels in critically ill patient groups, especially when pleural effusion is present. The findings demonstrate that albumin levels are very important in critically ill patients, especially those presenting with pleural effusion. This indicates that albumin may serve as a significant covariate influencing outcomes in biomarker analysis. Such advanced statistical adjustments are of critical importance in preventing the spurious correlations frequently encountered in biomarker studies. Additionally, the association between the HLR and both the length of hospital stay and the requirement for intensive care supports the premise that this ratio may predict not only mortality but also the intensity of healthcare resource utilization. In this study, HLR values also demonstrated significant differences based on the patients’ admission status at the time of initial presentation; lower values were noted in patients requiring hospital admission, thereby contributing to the clinical decision-making process and further supporting our hypothesis. When HLR levels were compared between ICU and general ward admissions, HLR levels were found to be statistically significantly lower in ICU patients (7.45 vs. 5.1; *p* < 0.001). While HLR differed notably across admission units, general ward vs. ICU, no significant correlation was observed between HLR and either ICU stay or total length of hospital stay (*p*-values > 0.05), indicating limitations in its utility as a biomarker for predicting hospitalization duration. The analysis of Glasgow Coma Scale (GCS) scores identified a moderate positive correlation with HLR (rho = 0.444, *p* < 0.001). HLR appears useful for identifying critically ill patients and guiding clinical decisions. Our results demonstrate that HLR is predictive for admission criteria, unit selection, clinical assessment, and survival prognosis.

A key strength of this study is its distinction as the first in the literature to examine the prognostic value of HLR specifically among pneumonia patients, with confirmation supported by adequate sampling power (88.5%). In addition to reporting the basic data, the predictive accuracy of the HLR was thoroughly assessed via advanced modeling (Models 1–3) and confounder analysis. However, certain limitations should be acknowledged. First, while the statistical significance (*p* < 0.001) and the consistency across different models (ROC and regression) support HLR as a promising predictor, these results should be interpreted with caution and validated in larger, prospective multicenter studies to obtain more precise effect size estimates. Also, the retrospective, single-center design of the research with no external validation requires careful consideration when extrapolating the findings. Furthermore, calculations relied exclusively on hemoglobin and lactate levels measured at presentation, without reflecting dynamic changes during treatment, such as lactate clearance or increases in hemoglobin following transfusion. Future prospective, multicenter research is needed to clarify the role of HLR in monitoring treatment response and evaluating its performance across different pneumonia etiologies (viral vs. bacterial). The strict nature of our patient exclusion criteria, combined with the exclusion of patients due to missing or erroneous (clotted blood) samples, may have introduced unintended biases. Consequently, the prevalence of positive cases in our sample may not fully reflect actual clinical figures. While we acknowledge this as a limitation, the results should be interpreted with caution.

The findings suggest that the hemoglobin-to-lactate ratio (HLR) is a cost-effective, accessible and clinically significant marker associated with mortality risk in pneumonia patients presenting to the emergency department. Particularly, HLR values below the threshold of 5.65 may indicate heightened risk, highlighting the importance of considering intensive care and close monitoring in these cases. Integrating HLR into established clinical scoring systems, such as CURB-65 and PSI, could enhance decision-making processes and warrant further investigation regarding its potential effect on pneumonia management outcomes. Prospective, multicenter studies are required for the generalizability of the results.

## 5. Conclusions

HLR serves as a potent, rapid, and cost-effective prognostic biomarker for patients presenting to the emergency department with pneumonia. An HLR value below the optimal cut-off of 5.65 is associated with a 10-fold increase in mortality risk and demonstrates predictive performance comparable to established clinical scoring systems such as CURB-65 and PSI. Furthermore, integrating HLR with the CURB-65 or PSI scoring improves diagnostic accuracy.

## Figures and Tables

**Table 1:** Descriptive statistical characteristics of quantitative data in the general sample.

Variable	Distribution ^†^
Age (years)	76 (18–100)
Respiratory rate (min^−1^)	20 (12–42)
SBP (mmHg)	111 (70–240)
DBP (mmHg)	70 (40–140)
Fever (°C)	37 (35.6–40)
Heart rate (min^−1^)	88 (20–170)
Saturation (Po) (%)	87 (40–98)
BUN (mg/dL)	18.5 (3.8–106.7)
Sodium (mEq/L)	137.6 (116.5–163.4)
Blood glucose (mg/dL)	132 (54.3–512.9)
Hematocrit (%)	37.6 ± 6.9
PH	7.4 (6.98–7.7)
PaO_2_ (mmHg)	42.2 (21.1–100.5)
SO_2_ (%)	63.4 (17.5–96.6)
pCO_2_ (mmHg)	40.2 (17–94.7)
Bicarbonate (mmol/L)	24.4 ± 5.5
Base excess (mmol/L)	−0.6 ± 5.5
WBC (K/µL)	11.5 (1.2–44)
Neutrophil (K/µL)	9.6 (0.7–87.8)
Hemoglobin (g/dL)	12.25 ± 2.37
RDW (%)	15.4 (12–50.6)
Lactate (mmol/L)	1.96 (0.7–7.7)
CRP (mg/L)	125.1 (0.7–426.4)
Procalcitonin (µg/L)	0.5 (0–97.2)
Albumin (g/L)	35.7 ± 6.5
Urea (mg/dL)	46.3 (9.4–266.7)
Creatinine (mg/dL)	1 (0.1–8.6)
GCS	15 (3–15)
RPE size (cm)	2 (0–8.5)
LPE size (cm)	2 (0–9)
LOHS (ICU) (days)	9 (1–84)
LOHS (Total) (days)	8 (1–109)
PSI	119.4 ± 44.5
CURB-65	1.8 ± 1.2
HLR	5.99 (1.54–19.28)

^†^ Data were expressed as mean ± standard deviation (SD) or median [range: minimum–maximum]) according to normal distribution characteristics. Abbreviations: LOHS = length of hospital stay (days), PSI = Pneumonia Severity Index, BUN = blood urea nitrogen, SBP = systolic blood pressure, DBP = diastolic blood pressure, GCS = Glasgow Coma Score, CRP = C-reactive protein, WBC = white blood cell, RPE = right pleural effusion, LPE = left pleural effusion, Po = pulse oximeter, HLR = Hemoglobin-to-lactate ratio.

**Table 2:** General distributional summary of gender, comorbidities, pneumonia and clinical characteristics of patients.

		Frequency (*N*)	Percentage (%)
Gender	Male	104	56.8%
	Female	79	43.2%
Comorbidity (overall)	No	33	18.0%
	Yes	150	82.0%
Nursing home resident	No	172	94.0%
	Yes	11	6.0%
Malignity	No	164	93.7%
	Yes	11	6.3%
Liver disease	No	166	90.7%
	Yes	17	9.3%
Congestive heart failure	No	154	84.2%
	Yes	29	15.8%
Cerebrovascular disease	No	158	86.3%
	Yes	25	13.7%
Renal failure	No	127	69.4%
	Yes	56	30.6%
Alzheimer’s disease/Dementia	No	140	76.5%
	Yes	43	23.5%
DM	No	130	71.0%
	Yes	53	29.0%
HT	No	103	56.3%
	Yes	80	43.7%
HL	No	165	90.2%
	Yes	18	9.8%
COPD	No	72	39.3%
	Yes	111	60.7%
CAD	No	159	86.9%
	Yes	24	13.1%
Confusion	No	135	73.8%
	Yes	48	26.2%
Presence of pleural effusion	No	125	68.3%
	Yes	58	31.7%
Lobar pneumonia	No	145	79.2%
	Yes	38	20.8%
Ground-glass opacity	No	78	42.6%
	Yes	105	57.4%
Air bronchogram	No	163	89.1%
	Yes	20	10.9%
Hospitalization at initial admission	No	32	17.5%
	Yes	151	82.5%
The unit of admission	Ward	57	37.7%
	ICU	94	62.3%
ES replacement therapy	No	168	91.8%
	Yes	15	8.2%
Vasopressor support	No	121	66.1%
	Yes	62	33.9%
Septicemia	No	44	24.0%
	Yes	139	76.0%
Dialysis treatment	No	179	97.8%
	Yes	4	2.2%
Overall survival (mortality)	Survived	123	67.2%
	Exitus	60	32.8%

Abbreviations: DM = diabetes mellitus, HT = hypertension, HL = hyperlipidemia, COPD = chronic obstructive pulmonary disease, CAD = coronary artery disease, ICU = intensive care unit, ES = erythrocyte suspension.

**Table 3:** General distributional comparison of gender, comorbidities, pneumonia and clinical characteristics among survival groups.

		Overall Survival		*p*-Value
		Survived	Exitus	
Gender	Male	72 (58.54%)	32 (53.33%)	0.505 ^a^
	Female	51 (41.46%)	28 (46.67%)	
Comorbidity (overall)	Yes	96 (78.05%)	54 (90%)	0.048 ^a^
Nursing home resident	Yes	6 (4.88%)	5 (8.33%)	0.345 ^b^
Malignity	Yes	9 (7.63%)	2 (3.51%)	0.507 ^a^
Liver disease	Yes	11 (8.94%)	6 (10%)	0.817 ^a^
Congestive heart failure	Yes	18 (14.63%)	11 (18.33%)	0.52 ^a^
Cerebrovascular disease	Yes	10 (8.13%)	15 (25%)	0.002 ^a^
Renal failure	Yes	29 (23.58%)	27 (45%)	0.003 ^a^
Alzheimer’s disease/Dementia	Yes	21 (17.07%)	22 (36.67%)	0.003 ^a^
DM	Yes	30 (24.39%)	23 (38.33%)	0.051 ^a^
HT	Yes	47 (38.21%)	33 (55%)	0.032 ^a^
HL	Yes	8 (6.5%)	10 (16.67%)	0.03 ^a^
COPD	Yes	69 (56.1%)	42 (70%)	0.071 ^a^
CAD	Yes	11 (8.94%)	13 (21.67%)	0.017 ^a^
Confusion	Yes	13 (10.57%)	35 (58.33%)	<0.001 ^a^
Presence of pleural effusion	Yes	30 (24.39%)	28 (46.67%)	0.002 ^a^
Lobar pneumonia	Yes	28 (22.76%)	10 (16.67%)	0.34 ^a^
Ground-glass opacity	Yes	62 (50.41%)	43 (71.67%)	0.006 ^a^
Air bronchogram	Yes	13 (10.57%)	7 (11.67%)	0.823 ^a^
Hospitalization at initial admission	Yes	91 (73.98%)	60 (100%)	<0.001 ^a^
The unit of admission	Ward	56 (61.54%)	1 (1.67%)	<0.001 ^a^
	ICU	35 (38.46%)	59 (98.33%)	
ES replacement therapy	Yes	4 (3.25%)	11 (18.33%)	<0.001 ^b^
Vasopressor support	Yes	6 (4.88%)	56 (93.33%)	<0.001 ^a^
Septicemia	Yes	81 (65.85%)	58 (96.67%)	<0.001 ^a^
Dialysis treatment	Yes	0 (0%)	4 (6.67%)	0.004 ^b^

Abbreviations: DM = diabetes mellitus, HT = hypertension, HL = hyperlipidemia, COPD = chronic obstructive pulmonary disease, CAD = coronary artery disease, ICU = intensive care unit, ES = erythrocyte suspension. ^a^ Pearson’s chi-squared test, ^b^ Fisher’s exact test.

**Table 4:** Comparison of laboratory results, vital signs, and clinical features among survival groups.

	Overall Survival		Overall	*p*
	Survived (*n* = 123, 67.2%)	Exitus (*n* = 60, 32.8%)		
Age (years)	74 (18–100)	83 (53–97)	76 (18–100)	<0.001 ^a^
Respiratory rate (min^−1^)	20 (12–40)	24.5 (12–42)	20 (12–42)	0.001 ^a^
SBP (mmHg)	120 (80–148)	110 (70–240)	111 (70–240)	0.081 ^a^
DBP (mmHg)	70 (50–95)	60 (40–140)	70 (40–140)	0.006 ^a^
Fever (°C)	37 (35.6–39)	36.7 (35.6–40)	37 (35.6–40)	0.281 ^a^
Heart rate (min^−1^)	85 (47–122)	94 (20–170)	88 (20–170)	0.002 ^a^
Saturation (Po) (%)	88 (47.9–98)	85.5 (40–97)	87 (40–98)	0.046 ^a^
PH	7.41 (7.18–7.7)	7.36 (6.98–7.52)	7.4 (6.98–7.7)	<0.001 ^a^
PaO_2_ (mmHg)	41.6 (21.1–100.5)	44.1 (22.9–90.9)	42.2 (21.1–100.5)	0.503 ^a^
SO_2_ (%)	63.1 (17.5–96.6)	69.1 (20.7–92.5)	63.4 (17.5–96.6)	0.330 ^a^
pCO2 (mmHg)	39.1 (17.4–94.7)	45.6 (17–73)	40.2 (17–94.7)	0.021 ^a^
Bicarbonate (mmol/L)	24.7 ± 4.8	23.8 ± 6.6	24.4 ± 5.5	0.386 ^b^
Base excess (mmol/L)	0.10 ± 4.2	−1.9 ± 7.4	−0.6 ± 5.5	0.067 ^b^
Lactate (mmol/L)	1.72 (0.7–5.9)	2.42 (1.1–7.7)	1.96 (0.7–7.7)	<0.001 ^a^
BUN (mg/dL)	16.2 (3.8–78.7)	32.5 (8.2–106.7)	18.5 (3.8–106.7)	<0.001 ^a^
Sodium (mEq/L)	137.4 (116.5–151.1)	137.9 (124.8–163.4)	137.6 (116.5–163.4)	0.339 ^a^
Blood glucose (mg/dL)	126.9 (60–512.9)	153.2 (54.3–512.1)	132 (54.3–512.9)	0.014 ^a^
Hemoglobin (g/dL)	12.74 ± 2.10	11.25 ± 2.60	12.25 ± 2.37	<0.001 ^b^
WBC (K/µL)	11.5 (2.7–34.8)	11.5 (1.2–44)	11.5 (1.2–44)	0.286 ^a^
Neutrophil (K/µL)	9.2 (1.5–87.8)	10.3 (0.7–87.8)	9.6 (0.7–87.8)	0.208 ^a^
RDW (%)	15.3 (12–48)	15.9 (13.5–50.6)	15.4 (12–50.6)	0.011 ^a^
CRP (mg/L)	116.5 (0.7–426.4)	155.9 (1.1–402.1)	125.1 (0.7–426.4)	0.023 ^a^
Procalcitonin (µg/L)	0.50 (0–97.2)	0.60 (0–21)	0.50 (0–97.2)	0.475 ^a^
Albumin (g/L)	37.8 ± 5.3	31.5 ± 6.8	35.7 ± 6.5	<0.001 ^b^
Urea (mg/dL)	40.6 (9.4–196.8)	81.3 (20.4–266.7)	46.3 (9.4–266.7)	<0.001 ^a^
Creatinine (mg/dL)	1.0 (0.1–4.1)	1.3 (0.2–8.6)	1 (0.1–8.6)	0.003 ^a^
GCS	15 (12–15)	14 (3–15)	15 (3–15)	<0.001 ^a^
RPE size (cm)	2 (0.5–8.5)	2 (0.5–5.4)	2 (0.5–8.5)	0.642 ^a^
LPE size (cm)	2.5 (1.5–4.5)	2.3 (0.5–9)	2.3 (0.5–9)	0.569 ^a^
LOHS (ICU) (days)	4 (1–26)	11 (1–84)	9 (1–84)	0.001 ^a^
LOHS (Total) (days)	7 (1–35)	12 (1–109)	8 (1–109)	0.003 ^a^
PSI	103.0 ± 37.0	153.1 ± 39.5	119.4 ± 44.5	<0.001 ^b^
CURB-65	1.4 ± 0.9	2.7 ± 1.1	1.8 ± 1.2	<0.001 ^b^
HLR	6.92 (2.54–19.28)	4.68 (1.54–13.0)	5.99 (1.54–19.28)	<0.001 ^a^

Data are expressed as mean ± standard deviation (SD) or median [range: minimum–maximum]) according to distributional characteristics. Abbreviations: LOHS = length of hospital stay (days), PSI = Pneumonia Severity Index, SBP = systolic blood pressure, DBP = diastolic blood pressure, GCS = Glasgow Coma Score, CRP = C-reactive protein, RPE = right pleural effusion, LPE = left pleural effusion, HLR = hemoglobin-to-lactate ratio, Po = pulse oximeter. ^a^ Mann–Whitney U test, ^b^ independent-samples *t*-test.

**Table 5:** Comparison of HLR values according to gender, comorbidities, hospitalization, clinical status, and pneumonia characteristics.

		HLR	*p*-Values *
Gender	Male	6.04 (1.54–16)	0.342
	Female	5.94 (1.6–19.28)	
Comorbidity (overall)	No	7.45 (3.35–16.21)	0.032
	Yes	5.8 (1.54–19.28)	
Presence of pleural effusion	No	6.5 (1.59–19.28)	0.013
	Yes	5.49 (1.54–14.94)	
Hospitalization at initial admission	No	6.86 (3.97–16)	0.018
	Yes	5.71 (1.54–19.28)	
The unit of admission	Ward	7.45 (2.67–19.28)	<0.001
	ICU	5.1 (1.54–13.09)	
Septicemia	No	6.78 (1.54–16.21)	0.069
	Yes	5.79 (1.59–19.28)	
Lobar pneumonia	No	6.02 (1.54–16.21)	0.938
	Yes	5.66 (1.62–19.28)	
Ground-glass opacity	No	6.82 (1.86–16.21)	0.012
	Yes	5.54 (1.54–19.28)	
Air bronchogram	No	6.18 (1.54–19.28)	0.36
	Yes	5.58 (1.62–10.9)	

Data are expressed as interquartile ranges median [range: minimum–maximum]). * Mann–Whitney U test.

**Table 6:** Comparison of HLR among pleural effusion groups with adjustment of albumin (covariate).

	**Pleural Effusion**			**ANCOVA ^†^**		
	No (*n* = 125, 68.3%)	Yes (*n* = 58, 31.7)				
	**Distribution**					
			***p*_1_**	***F***	***p*_2_**	***η_p_* ^2^**
HLR *	6.5 (1.59–19.28)	5.49 (1.54–14.94)	0.013 ^a^	1.568	0.212 ^b^	0.009

* Logarithmic transform has been performed in order to convert skewed variables into a normal distribution pattern. Abbreviations: HLR = hemoglobin-to-lactate ratio, *p*_1_ = *p*-value before ANCOVA analysis, *p*_2_ = *p*-value after adjustment (ANCOVA analysis). ^†^ Covariates used for adjustment in the model: albumin. ^a^ Mann–Whitney U test, ^b^ ANCOVA analysis.

**Table 7:** Investigation of the impact profiles of variables on mortality through univariate and multivariate logistic regression (LR) analysis.

LR Analysis								
	Univariate LR						Multivariate LR	
Variables ^†^	Nagelkerke R^2^	B	*p*	OR	95% CI		Nagelkerke R^2^ = 0.483	
					Lower Limit	Upper Limit	*p*	OR (95% CI)
Age (years)								
<77.0 (ref)	–	–	–	–	–	–	–	–
≥77.0	0.127	1.362	<0.001	3.91	2.02	7.57	0.002	4.13 (1.70–10.0)
Gender ^§^								
Male (ref)	–	–	–	–	–	–	–	–
Female	0.003	0.211	0.505	1.24	0.66	2.30	0.898	0.95 (0.43–2.12)
Comorbidity (overall) ^§^								
No (ref)	–	–	–	–	–	–	–	–
Yes	0.032	0.929	0.054	2.53	0.98	6.52	0.39	1.70 (0.51–5.68)
CRP								
<213.5 (ref)	–	–	–	–	–	–	–	–
≥213.5	0.059	0.994	0.005	2.70	1.36	5.39	0.089	2.14 (0.89–5.13)
CURB-65								
<2.0 (ref)	–	–	–	–	–	–	–	–
≥2.0	0.254	2.217	<0.001	9.18	4.02	20.97	0.04	2.77 (1.05–7.35)
PSI *								
<133.0 (ref)	–	–	–	–	–	–	–	–
≥133.0	0.332	2.428	<0.001	11.33	5.49	23.40	–	–
HLR								
>5.65 (ref)	–	–	–	–	–	–	–	–
≤5.65	0.342	2.497	<0.001	12.15	5.65	26.13	<0.001	10.01 (4.15–24.19)

Reference category of analysis: Survival group. Abbreviations: CI = Confidence Interval, OR = odds ratio, LR = logistic regression, ref = reference subcategory. ^†^ The cut-off values were obtained from ROC analysis, based on Youden’s J index. * Due to the fact that the PSI score is calculated based on age, gender, hematocrit and comorbidities, it was excluded from the multivariate model to avoid the violation of multicollinearity assumption. ^§^ Although these variables did not reach statistical significance in univariate analyses, it was included in the multivariate models for adjustment purposes as a potential confounder.

**Table 8:** Investigation of the predictive features of quantitative variables and combined models on mortality using ROC analysis.

Variables	AUC (95% CI)	Cut-Off ^†^	*p*-Value	Sensitivity (%)	Specificity (%)	NPV (%)	PPV (%)
Age (years)	0.704 (0.625–0.784)	≥77.0	<0.001	70.0%	62.6%	81.1%	47.7%
Lactate (mmol/L)	0.728 (0.652–0.803)	≥2.1	<0.001	66.7%	70.7%	81.3%	52.6%
Hemoglobin (g/dL)	0.668 (0.581–0.755)	≤11.15	<0.001	51.7%	78.9%	77.0%	54.4%
CRP (mg/L)	0.603 (0.512–0.695)	≥213.5	0.027	38.3%	81.3%	73.0%	50.0%
PSI	0.829 (0.768–0.889)	≥133.0	<0.001	70.0%	82.9%	85.0%	66.7%
CURB-65	0.807 (0.742–0.871)	≥2.0	<0.001	86.7%	58.5%	90.0%	50.5%
HLR	0.802 (0.733–0.871)	≤5.65	<0.001	81.7%	74.0%	89.2%	60.5%
Model 1 (HLR + CRP)	0.805 (0.736–0.874)	–	<0.001	80.0	76.4	88.7	62.3
Model 2 (HLR + CURB-65)	85.7 (79.6–91.7)	–	<0.001	75.0	84.6	87.4	70.3
Model 3 (HLR + PSI)	85.4 (79.7–91.1)	–	<0.001	78.3	83.7	70.1	70.1

Abbreviations: CRP = C-reactive protein, PSI = Pneumonia Severity Index, HLR = hemoglobin-to-lactate ratio, AUC = area under the curve, NPV = negative predictive value, PPV = positive predictive value, ROC = receiver operating characteristic. ^†^ Based on Youden’s J index. Reference Category: Survival group.

**Table 9:** Pairwise comparison of the overall prediction characteristics of the models.

Pairwise Comparisons of Models	Asymptotic		AUC Difference	Standard Error Difference	Asymptotic 95% Confidence Interval	
	z	*p*-Value			Lower Bound	Upper Bound
Model 1–Model 2	−2.081	0.037	−0.051	0.254	−0.099	−0.003
Model 1–Model 3	−1.903	0.057	−0.049	0.251	−0.099	0.001
Model 2–Model 3	0.148	0.882	0.003	0.242	−0.031	0.037

Model 1 = HLR + CRP, Model 2 = HLR + CURB-65, Model 3 = HLR + PSI. Pairwise comparisons are carried out using DeLong’s method.

## Data Availability

The data presented in this study are available on request from the corresponding author. The data are not publicly available due to privacy and ethical reasons.
